# Effect of acuity level and patient characteristics on bowel preparation quality: a retrospective cohort study of inpatient colonoscopies

**DOI:** 10.1186/s12876-023-02751-1

**Published:** 2023-04-15

**Authors:** Christopher Kabir, Mariani Salazar Leon, Cindy Ndiaye, Michael Flicker

**Affiliations:** Advocate Illinois Masonic Medical Center, Advocate Aurora Research Institute, Advocate Aurora Health, Center for Education, 836 W Wellington Ave, #2025 Chicago, IL 60657 USA

**Keywords:** Preoperative care (MeSH), Colonoscopies (MeSH), Hospitalization (MeSH), Bowel preparation quality

## Abstract

**Background and aims:**

Colonoscopy is the primary method to detect mucosal abnormalities in the colon, rectum, and terminal ileum. Inadequate bowel preparation is a common problem and can impede successful visualization during colonoscopy. Although studies identified hospitalization as a predictor of inadequate bowel preparation, acuity of care vary greatly within this patient population. The current study aims to examine the effect of patient characteristics and care level predictors on inadequate bowel preparation quality within the inpatient setting.

**Methods:**

This retrospective study was conducted in a single urban level 1 trauma medical center and included adult patients undergoing diagnostic colonoscopy while admitted in the hospital from January 1, 2015 to June 30, 2020. We examined the level of inpatient care between the General Medical Floor (GMF), Intensive Care Units (ICU) and Telemetry Unit (TU) and assessed this association with bowel preparation quality, adjusting for known and unknown predictors.

**Results:**

Of 538 patients undergoing colonoscopy, 47.4% were admitted into TU, 43.7% into GMF and 8.9% into ICU. For the entire sample, 72.7% of patients achieved good or excellent preparation and quality of bowel preparation differed by care level (*P* = 0.01). Patients from the critical care units were less likely to achieve adequate bowel preparation when compared to GMF (Odds Ratio [OR] 0.36; 95% Confidence Interval [CI] 0.17,0.77), after adjusting for patient characteristics, medications, physical status, and preparation regimen. No significant difference in Bowel Preparation Quality (BPQ) was identified between patients from GMF and TU (OR 0.96; 95%CI 0.61, 1.52). Furthermore, adequate BPQ was associated with withdrawal time and cecal intubation, but not higher adenoma detection rates.

**Conclusions:**

Results suggest the ICU setting is an independent predictor for inadequate bowel preparation and patients with prior opioid and laxative use may be more likely to have inadequate bowel preparation in the hospital. Future interventions should prioritize preprocedural clinician meetings for critical care unit patients, including a more detailed readiness assessment and thorough medication history.

## Introduction

Colonoscopies are the gold standard for diagnosing colorectal cancer, the second most common cause of cancer death in the United States of America. Colorectal cancer, while currently declining in ages 65 years and older, is increasing in incidence among younger Americans [1]. In response, the U.S. Multi-Society Task Force on Colorectal Cancer revised its age recommendations to begin colorectal cancer screening five years earlier, from 50 to 45 years of age, and reaffirmed the importance of early diagnosis, including prompt assessment of symptoms such as blood loss or anemia [2]. Diagnostic efficacy, however, is dependent upon Bowel Preparation Quality (BPQ), which is often categorized as Inadequate Bowel Preparation (IBP) or Adequate Bowel Preparation (ABP).

It is well established that ABP is difficult to achieve in hospitalized patients and inpatient status is consistently reported as a strong independent predictor of IBP. Research shows that 67% (60–75%) of hospitalized patients attain ABP prior to colonoscopy, and ABP remains up to two times more likely (OR [Odds Ratio] 2.02; 95%CI [Confidence Interval] 1.88, 2.16) among outpatients when compared to inpatients [3–6]. Lower ABP rates among hospitalized patients have negative consequences for the patient and may result in reduced diagnostic yield, increased hospital costs, and prolonged length of stay [7, 8].

Bowel preparation quality has been studied in many healthcare settings, identifying a variety of clinical and patient predictors of IBP, including: older age [4, 5, 7, 9, 10], male gender [4–6, 9, 11], medications such as tricyclic antidepressants or opiates [5, 6, 8], constipation [5, 6, 12], afternoon procedure times [6, 8, 9, 13], prolonged hospitalization before colonoscopy [12], preprocedural physical fitness [8, 12], type of bowel preparation regimen [3, 12, 14], and significant comorbidities such as diabetes mellitus [5, 11, 12]. Despite robust evidence that outpatients achieve higher quality of bowel preparation when compared to inpatients, no study has investigated patient disposition within a hospital as an opportunity to identify modifiable factors and setting-based interventions to improve BPQ.

A clear understanding of patient and care level variables within the hospital can identify patients who may benefit from enhanced pre-colonoscopy readiness assessments or different bowel preparation protocols. The aim of this study was to investigate the association between the quality of bowel preparation and predictors stratified by acuity level of inpatient care between the General Medical Floor (GMF), Telemetry Unit (TU), and Intensive Care Units (ICU). We hypothesized that ICU patients had worse quality of bowel preparation. Secondary objectives were to evaluate adequate bowel preparation related to quality metrics, colonoscopic findings, length of stay prior to colonoscopy, and time of colonoscopy.

## Methods

### Participants and study design

This retrospective study was conducted at a level 1 trauma medical center in metropolitan Chicago, Illinois, United States of America. From January 1, 2015 to June 30, 2020, electronic medical record data was collected from patients ≥ 18 years of age who underwent a colonoscopy while admitted in the hospital. Eligible patients received bowel preparation with polyethylene glycol (PEG) based solution, including patients with additional laxatives, magnesium citrate, or enemas prior to colonoscopy. Patients were excluded if they completed bowel preparation as outpatients, had a history of bowel surgery, underwent sigmoidoscopy or enema only preparation, were pregnant, or were non-compliant with the bowel preparation. This study adhered to the reporting guidelines of the STROBE checklist used for observational studies.

### Variables

Level of care was recorded at the time of colonoscopy preparation. The nurse-to-patient ratio for each care level was 1:8 in GMF, 1:4 in TU, and 1:1 or 1:2 in ICU. Data was extracted from physician and nursing notes at the time of colonoscopy. Patient information included: demographics, prior abdominopelvic surgery, history of constipation and use of laxatives, medications, comorbidities, and neurologic conditions. Preprocedural fitness was defined by American Society of Anesthesiologists (ASA) class: I, normal healthy patient; II, patient with mild systemic disease; III, patient with severe systemic disease, and; IV, patient with severe systemic disease that is a constant threat to life [15].

Indication for colonoscopy was classified as anemia or blood loss, abnormal abdominal imaging, inflammatory bowel disease, diarrhea, constipation, abdominal pain, weight loss, or unknown. Periprocedural and quality variables collected included: morning or afternoon colonoscopy time, successful cecal intubation, withdrawal time and colonoscopy findings described as small polyp < 1 cm, large polyp ≥ 1 cm or malignant mass.

Bowel preparation quality was assessed by the endoscopist using Provation software and categorized in a traditional scale as: poor (large amount of fecal residue precluding a complete examination), fair (moderate amount of stool cleared with suctioning permitting an adequate evaluation of entire colonic mucosa), good (small amount of turbid fluid or feces not interfering with the examination), and excellent (small amount of clear liquid with a clear mucosa seen) [16]. Bowel preparation regimen was categorized as standard preparation with 4L PEG in a one-time dose, split preparation, or combination with magnesium citrate, enema, or stimulant laxatives.

### Statistical analysis

Descriptive statistics were reported as means with standard deviations for normally distributed variables or medians with interquartile ranges for non-normally distributed continuous variables. Counts and percentages were reported for categorical variables. Comparison between units were assessed using overall *x*^2^, ANOVA for normally distributed variables or Kruskal–Wallis tests for non-normally distributed variables. Adequate versus inadequate bowel preparation was tested using the Fisher’s exact or *x*^2^ test, Student’s T-test, or Mann–Whitney U test. The final multivariable logistic regression model was selected using a forward stepwise approach to account for clinically significant and confounding variables on adequacy of bowel preparation. Regression results were reported as Odds Ratios and 95% Confidence Intervals. Statistical significance was determined by 2-sided tests and α = 0.05. Breslow-Day tests examined effect modification and collinearity was determined by variance inflation factor < 10 and tolerance > 0.1. Analysis was conducted using SAS 9.4 (SAS Institute Inc., Cary, NC, USA).

## Results

### Patient characteristics

The study identified 609 patients who underwent colonoscopies with bowel preparation documentation. Seventy-one patients were excluded: 39 had prior bowel surgery, 10 had Ogilvie’s syndrome, four had volvulus, seven had outpatient bowel preparation, four had incomplete documentation, three did not have the colonoscopy, and four had enema-only preparation.

The remaining 538 patients were included in analysis and data from the first procedure were collected for 14 patients who required repeat colonoscopies due to poor bowel preparation (eight from GMF, four from TU, and two from ICU). The final analytic sample had a median age of 67 years (interquartile range [IQR]: 51–79), was comprised of 250 (46.47%) females, and represented a diverse patient population with 120 (22.30%) Blacks, 158 (29.37%) Hispanic/ Latinos, 32 (5.95%) Asians, and 224 (41.64%) Whites.

The majority of patients, 419 (77.88%), received standard PEG 4L regimen regardless of care level. Age was associated with care level, and older patients were admitted to higher acuities of care; the median ages in years and interquartile ranges by care level were 58 (40–73) in GMF, 70 (62–80) in TU, and 72 (62–80) in ICU. The median hospital length of stay prior to colonoscopy was 2 days (Interquartile range = 2). Descriptive statistics for each care level are reported in Table 1, in which differences were identified in diabetes mellitus and ASA classification. Indication for colonoscopy also differed by care level, with a lower proportion of patients with blood loss or anemia in the GMF when compared to TU and ICU (54.0% vs. 92.2% vs. 95.8%, respectively; *P* < 0.001).

**Table 1:** Patient and clinical characteristics stratified by acuity level

	General Medical Floor	Telemetry Unit	Intensive Care Unit
	*n* = 235	*n* = 255	*n* = 48
Age (years)*^,a^, median (IQR)	58.00 (40.00–73.00)	58.00 (40.00–73.00)	72.00 (62.00–79.50)
Female gender	108 (45.96%)	125 (49.02%)	17 (35.42%)
Race/ ethnicity			
White	109 (46.38%)	97 (38.04%)	18 (37.50%)
Black	50 (21.28%)	58 (22.75%)	12 (25.00%)
Hispanic/Latino	56 (23.83%)	88 (34.51%)	14 (29.17%)
Asian	16 (6.81%)	12 (4.71%)	4 (8.33%)
Unknown/Other	4 (1.70%)	0 (0.0%)	0 (0.0%)
Body Mass Index (kg/m^2^)			
Underweight (< 18.5)	15 (6.49%)	14 (5.51%)	3 (6.25%)
Normal (18.5–24.9)	79 (34.20%)	60 (23.62%)	14 (29.17%)
Overweight (25.0–29.9)	89 (38.53%)	105 (41.34%)	22 (45.83%)
Obese (≥ 30.0)	48 (20.78%)	75 (29.53%)	9 (18.75%)
History of abdominal or pelvic surgery	69 (29.36%)	82 (32.16%)	12 (25.00%)
Prior use of laxatives	23 (9.79%)	29 (11.46%)	5 (10.42%)
Current medications			
None	186 (79.15%)	182 (71.37%)	35 (72.92%)
Opioids	23 (9.79%)	26 (10.20%)	9 (18.75%)
TCA	3 (1.28%)	4 (1.57%)	0 (0.0%)
Diltiazem	0 (0.0%)	10 (3.92%)	1 (2.08%)
Iron	19 (8.09%)	28 (10.98%)	3 (6.25%)
Multiple	4 (1.71%)	5 (1.96%)	0 (0.0%)
Neurologic condition*			
None	204 (86.81%)	220 (86.27%)	39 (81.25%)
Stroke	8 (3.40%)	18 (7.06%)	2 (4.17%)
Dementia	13 (5.53%)	5 (1.96%)	3 (6.25%)
Paraplegia	2 (0.85%)	0 (0.0%)	0 (0.0%)
Parkinson’s disease	0 (0.0%)	2 (0.78%)	0 (0.0%)
Other	4 (1.70%)	8 (3.14%)	2 (4.17%)
Multiple conditions	4 (1.70%)	2 (0.78%)	1 (2.08%)
Intubated	0 (0.0%)	0 (0.0%)	1 (2.08%)
Any neurologic condition	31 (13.19%)	35 (13.73%)	9 (18.75%)
Diabetes mellitus diagnosis*	29 (12.34%)	65 (25.49%)	12 (25.00%)
Bowel preparation regimen			
PEG 4L	182 (77.45%)	202 (79.22%)	35 (72.92%)
PEG split	19 (8.09%)	28 (10.98%)	6 (12.50%)
PEG + magnesium citrate	8 (3.40%)	2 (0.78%)	1 (2.08%)
PEG + enema	4 (1.70%)	1 (0.39%)	2 (4.17%)
PEG + laxative	1 (0.43%)	2 (0.78%)	0 (0.0%)
Unknown	18 (7.66%)	14 (5.49%)	4 (8.33%)
Combination	3 (1.28%)	6 (2.35%)	0 (0.0%)
ASA risk classification*			
I	22 (9.40%)	7 (2.76%)	0 (0.0%)
II	155 (66.24%)	160 (62.99%)	5 (10.42%)
III	51 (21.79%)	76 (29.92%)	33 (68.75%)
IV	6 (2.56%)	11 (4.33%)	10 (20.83%)
Indication for colonoscopy*			
Anemia or blood loss	127 (54.04%)	235 (92.16%)	46 (95.83%)
IBD	24 (10.21%)	2 (0.78%)	46 (95.83%)
IBD	24 (10.21%)	2 (0.78%)	0 (0.0%)
Diarrhea and pain	13 (5.53%)	1 (0.39%)	0 (0.0%)
Diarrhea	19 (8.09%)	8 (3.14%)	1 (2.08%)
Constipation	4 (1.70%)	2 (0.78%)	0 (0.0%)
Abdominal pain	27 (11.49%)	3 (1.18%)	0 (0.0%)
Abnormal CT	5 (2.13%)	0 (0.0%)	1 (2.08%)
Weight loss	6 (2.55%)	3 (1.18%)	0 (0.0%)
Not documented/ unknown	10 (4.26%)	1 (0.39%)	0 (0.0%)
Afternoon procedure time	172 (73.19%)	192 (75.29%)	40 (83.33%)
Cecal Intubation*	206 (87.66%)	242 (94.90%)	40 (83.33%)
Withdrawal time ≥ 6 min	206 (87.66%)	228 (89.41%)	43 (89.58%)
≥ 7 days hospitalized before procedure	14 (5.96%)	10 (3.92%)	5 (10.42%)

*Abbreviations*: *TCA* Tricyclic antidepressants, *PEG* Polyethylene glycol, *ASA* American Society of Anesthesiologists, *IBD* Inflammatory bowel disease, *CT* Computerized tomography

^a^Analysis was conducted by the Kruskall-Wallis test. Variables and number of missing values are: BMI, 5; ASA, 2; Prior use of laxatives, 2

^*^Results indicate statistical significance at *P* < 0.05

### Bowel preparation quality

**Fig. 1:**
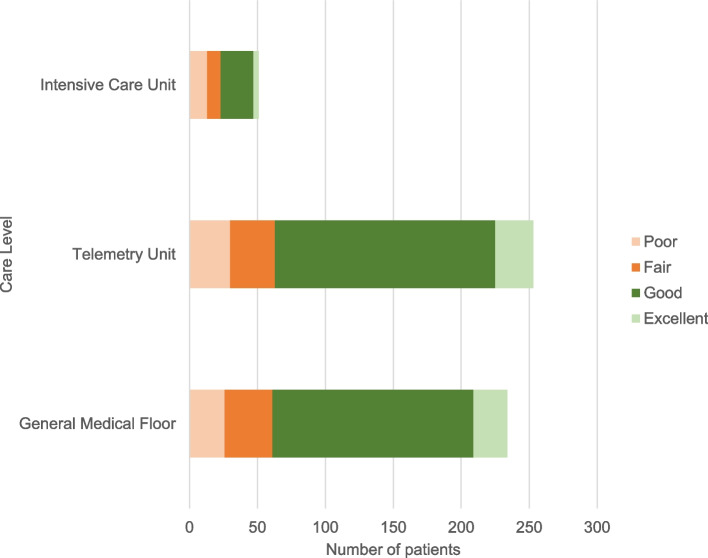
Distribution of bowel preparation quality categories by inpatient setting

Overall, 391 (72.7%) patients achieved ABP, among which 57 (14.58%) were classified as excellent and 334 (85.42%) as good. Adequate bowel preparation was achieved in 173 (73.62%) of patients in GMF, 191 (74.90%) in TU, and 27 (56.25%) in ICU (Fig. 1).

Quality of bowel preparation was found to differ by care level and the intensive care unit was identified as an independent risk factor for inadequate bowel preparation (*P* = 0.027). After adjusting for statistically and clinically significant variables (refer to Table 2), ICU patients had 64% lower odds of adequate bowel preparation in comparison to GMF (adjusted Odds Ratio [OR] 0.36; 95% Confidence Interval [CI] 0.17,0.77) and no significant difference in BPQ was identified between GMF and TU (OR 0.96; 95%CI 0.61, 1.52).

**Table 2:** Results for unadjusted and adjusted logistic regression models for adequate bowel preparation

Variable	Odds Ratio	Unadjusted (95% CI)	Odds Ratio	Adjusted (95% CI)
Acuity level*				
GMF	Ref		Ref	
TU	1.07	0.71–1.60	0.96	0.61–1.52
ICU	0.46	0.24–0.87	0.36	0.17–0.77
Age	0.99	0.99–1.01	0.99	0.99–1.01
Gender				
Female	Ref		Ref	
Male	0.65	0.44–0.96	0.58	0.382–0.88
Race/ ethnicity				
White	Ref		Ref	
Black	0.62	0.39–1.00	0.65	0.40–1.07
Hispanic/Latino	1.47	0.91–2.40	1.47	0.87–2.48
Asian	1.34	0.55–3.25	1.58	0.62–4.04
Unknown/Other	0.37	0.05–2.72	0.49	0.06–4.06
Prior laxative use*	0.47	0.27–0.83	0.45	0.24–0.86
Bowel prep regimen				
PEG 4 L	Ref		Ref	
PEG split	1.81	0.85–3.82	1.95	0.89–4.29
Other	0.61	0.35–1.04	0.58	0.33–1.04
ASA				
ASA I	Ref		Ref	
ASA II	1.27	0.57–2.81	1.37	0.56–3.32
ASA III	1.63	0.70–3.81	2.30	0.86–6.15
ASA IV	1.21	0.38–3.88	1.54	0.44–5.43
Prior opioid use*	0.38	0.22–0.67	0.49	0.26–0.91

*Abbreviations*: *CI* Confidence interval, *GMF* General medical floor, *TU* Telemetry unit, *ICU* Intensive care unit, *PEG* Polyethylene glycol, *ASA* American Society of Anesthesiologists

^*^Results indicate statistical significance at *P* < 0.05

Patient characteristics, medical history, and periprocedural variables were tested for association with ABP and acuity level. Patients with lower functioning physical status defined by ASA categories were admitted to higher acuities of care, although preprocedural physical status was not associated with BPQ.

Among covariates, previous history of constipation and laxative use, history of opioid use, and male gender were identified as independent predictors of inadequate bowel preparation quality. Laxative use was strongly associated with BPQ. Upon assessment of laxative use, statistical collinearity was identified between laxative use and constipation, with tolerance of 0.08 and VIF of 12.99. Laxative use was selected in the final model, as this variable can describe patients with constipation severe enough to require medicinal intervention. Prior opioid medication was also identified as an independent predictor of inadequate preparation (OR 0.38; 95%CI 0.23–0.68). For the final model presented in Table 2, all independent variables were tested for collinearity and exhibited adequate tolerance and variance inflation factors.

### Acuity of care and predictors of adequate bowel preparation

A gender effect was identified in the total sample. Among 147 hospitalized patients with IBP, 57 (38.8%) were females and 90 (61.2%) were males. After adjusting for covariates, males were at 0.42 lower odds (OR 0.58; 95%CI 0.38, 0.88) than females to have adequate bowel preparation. Within ad hoc sub-group analysis of the GMF care level, males were substantially more likely than females to have IBP in GMF (69.4% vs. 30.7%; *P* = 0.005).

History of opioid use remained associated with lower likelihood of adequate preparation after adjustment (OR 0.49; 95%CI 0.26, 0.91). Exploratory analysis of the 58 inpatients who had taken opioids showed that 31 (53.5%) had ABP and 27 (46.6%) had IBP; furthermore, lower proportions of patients with ABP had prior opioids when compared to IBP within units for TU (42.3% ABP vs. 57.7% IBP; *P* < 0.001) and ICU (33.3% vs. 66.6%; *P* = 0.124).

Although statistically non-significant, trends for IBP were identified with non-standard bowel preparations and African American ethnicity. We did not find significant associations between BPQ and the following covariates: age, prior abdominopelvic surgery, diabetes, ASA classification status, or procedural indication (Table 2).

### Secondary results

For secondary outcomes depicted in Table 3, no evidence was identified for associations of ABP with neurological diagnosis, length of preprocedural stay, colonoscopy time, or colonoscopic findings for small polyps, large polyps, or masses. The median length of stay before colonoscopy was two days (Interquartile range = 2), and this was not associated with quality of bowel preparation (*P* = 0.67). No statistically significant differences in BPQ were identified when examining the presence of any neurologic condition, despite fewer neurologic diagnoses in GMF and TU in comparison to ICU (13.2% vs. 13.7% vs. 18.8%, respectively; *P* = 0.59). Patients were more likely to be hospitalized for ≥ 7 days in ICU than GMF or TU (10.4% vs. 6.0% vs. 3.9%, respectively; *P* = 0.16), and this was not associated with BPQ. There was no significant difference between the quality of bowel preparation and colonoscopy time (*P* = 0.59), and performance of colonoscopies in the later afternoon suggested a trend toward improved bowel preparation.

**Table 3:** Secondary results stratified by bowel preparation quality

	Total	Adequate	Inadequate	*P*-Value
	*N* = 538	*n* = 391	*n* = 147	
Days in hospital prior to colonoscopy				
> 7 days	29 (5.26%)	19 (4.76%)	10 (6.58%)	0.3933
< 7 days	522 (94.74%)	380 (95.24%)	142 (93.42%)	
Any neurological diagnosis	75 (13.94%)	52 (13.30%)	23 (15.65%)	0.4837
Colonoscopy time				0.5934
Afternoon	404 (75.09%)	296 (75.70%)	108 (73.47%)	
Morning	134 (24.91%)	95 (24.30%)	39 (26.53%)	
Cecal intubation*	488 (90.71%)	371 (94.88%)	117 (79.59%)	< 0.0001
Withdrawal time ≥ 6 min*	477 (88.66%)	362 (92.58%)	115 (78.23%)	< 0.0001
Abnormal findings				
Small polyp	167 (31.10%)	125 (31.97%)	42 (28.77%)	0.4757
Large polyp	46 (8.57%)	33 (8.44%)	13 (8.90%)	0.8642
Mass	37 (6.89%)	29 (7.42%)	8 (5.48%)	0.4303
At least 1 finding^a^	211 (39.29%)	159 (40.66%)	52 (35.62%)	0.2865

^a^At least 1 finding was defined as a composite variable to include patients with any one or combination of detected small polyp, large polyp or mass

^*^Results indicate statistical significance at *P* < 0.05. 1 patient was missing colonoscopic findings

Quality metric variables were associated with BPQ, with cecal intubation and withdrawal time ≥ 6 min more likely to be reached in patients with ABP. Overall, cecal intubation was reached in 488 (90.8%) of cases and 477 (88.7%) had a withdrawal time ≥ 6 min. Patients with IBP were less likely to reach the cecum when compared with patients with ABP (79.6% vs. 94.9%, *P* > 0.001), and 43 (83.3%) of ICU patients reached cecal intubation. No statistically significant difference was found between finding polyps of any size and BPQ (*P* = 0.287) (Table 3).

## Discussion

The current study provides greater understanding of hospitalized patients undergoing colonoscopy. Consistent with prior investigations, history of opiates [5, 6, 8], constipation [5, 6, 12], and male gender [4–6, 9, 11] were identified as independent predictors of IBP*.* Contrary to literature, age was not identified as a risk factor for IBP [10]. A possible explanation for this may be that difficulty following instructions and older age may contribute less to BPQ in inpatient procedures and more in outpatient colonoscopies. Within the hospital, prior usage of opioids and laxatives or history of constipation may be more predictive of inpatient cleansing difficulties.

While the effectiveness of different bowel preparation regimens was considered, the majority of the analytic sample was treated with a 4L PEG regimen and we did not have sufficient statistical power to test for differences in more granularity. However, trends were identified toward greater effectiveness of split vs. 4L PEG regimen, and greater effectiveness of 4L PEG regimen vs. alternative combination regimens. Secondary results indicate that ABP was associated with a higher likelihood of reaching the cecum, but not related to increased adenoma or cancer detection.

This retrospective study had other limitations, including variability in skill and experience between colonoscopists, subjective measurement of BPQ and ASA categorization by specialists, and lack of standardized pre-procedure and patient compliance documentation. Although BPQ classification was entered using a simplified 4-point scale incorporating both cleanliness and volume, the study scale does not distinguish between colon segments to the extent of other tools, such as the Ottawa or Boston bowel preparation scales [16, 17]. Lastly, patients with complex care needs were transferred into multiple units, which may lead to unobserved care differences not captured by the current study. Of note, only one intubated patient underwent colonoscopy; therefore, we cannot attribute the poor quality of bowel preparation in the ICU to intubated status or sedation. Despite these limitations, this unique study examines patients by unit at time of colonoscopy and adjusts for physical status and known and unknown confounders, such as bowel preparation regimen and prior medication use.

While research shows promise for outpatient, screening, and patient-level interventions to improve ABP, future studies are needed to evaluate hospital and clinician-level interventions in higher risk populations [3, 18]. One potential solution to improve ABP in the hospital setting is to prioritize preprocedural clinician meetings [12]. Ongoing clinical education is also recommended and can include hospital lectures and initiatives on prompt symptom identification, appropriate referrals, standardized order sets, and improved preprocedural readiness assessments [19–21]. Further research in this topic is warranted and these results suggest interventions may be increasingly effective when identifying hospitalized patients with a history of opioid use, laxative use, or constipation.

In conclusion, this investigation provides a detailed examination of the inpatient care setting, where patients in critical care are more likely to have IBP and may be most likely to benefit from enhanced readiness assessment and thorough patient medical histories. Enhanced clinical education in the inpatient setting remains beneficial and future protocols should prioritize timing and administration of bowel preparation to improve ABP in higher acuity settings.

## Data Availability

The dataset analyzed for the current study is available from the corresponding author on reasonable request and is contingent upon institutional approvals that apply to the availability of data.

## Abbreviations

GMF: General Medical Floor
ICU: Intensive Care Units
TU: Telemetry Unit
OR: Odds Ratio
CI: Confidence Interval
BPQ: Bowel Preparation Quality
IBP: Inadequate Bowel Preparation
ABP: Adequate Bowel Preparation
PEG: Polyethylene Glycol
ASA: American Society of Anesthesiologists
IQR: Interquartile Range

## References

[1] Siegel RL, Miller KD, Goding Sauer A (2020). Colorectal cancer statistics, 2020. CA Cancer J Clin.

[2] American Gastroenterological Association. Statement from the U.S. Multisociety Task Force on Colorectal Cancer. 2018; https://gastro.org/press-releases/statement-from-the-u-s-multisociety-task-force-on-colorectal-cancer/. Accessed 25 Aug 2021.

[3] Gkolfakis P, Tziatzios G, Papanikolaou IS, Triantafyllou K (2019). Strategies to improve inpatients’ quality of bowel preparation for colonoscopy: a systematic review and meta-analysis. Gastroenterol Res Pract.

[4] Baker FA, Mari A, Nafrin S (2019). Predictors and colonoscopy outcomes of inadequate bowel cleansing: a 10-year experience in 28,725 patients. Ann Gastroenterol.

[5] Mahmood S, Farooqui SM, Madhoun MF (2018). Predictors of inadequate bowel preparation for colonoscopy: a systematic review and meta-analysis. Eur J Gastroenterol Hepatol.

[6] Ness RM, Manam R, Hoen H, Chalasani N (2001). Predictors of inadequate bowel preparation for colonoscopy. Am J Gastroenterol.

[7] Froehlich F, Wietlisbach V, Gonvers JJ, Burnand B, Vader JP (2005). Impact of colonic cleansing on quality and diagnostic yield of colonoscopy: the European Panel of Appropriateness of Gastrointestinal Endoscopy European multicenter study. Gastrointest Endosc.

[8] Yadlapati R, Johnston ER, Gregory DL, Ciolino JD, Cooper A, Keswani RN (2015). Predictors of inadequate inpatient colonoscopy preparation and its association with hospital length of stay and costs. Dig Dis Sci.

[9] Lebwohl B, Wang TC, Neugut AI (2010). Socioeconomic and other predictors of colonoscopy preparation quality. Dig Dis Sci.

[10] McNabb-Baltar J, Dorreen A, Al Dhahab H (2016). Age is the only predictor of poor bowel preparation in the hospitalized patient. Can J Gastroenterol Hepatol.

[11] Zad M, Do CN, Heffernan A, Johnston L, Al-Ansari M (2020). Factors affecting bowel preparation adequacy and procedural time. JGH Open.

[12] Fuccio L, Frazzoni L, Spada C (2021). Factors that affect adequacy of colon cleansing for colonoscopy in hospitalized patients. Clin Gastroenterol Hepatol.

[13] Jawa H, Mosli M, Alsamadani W (2017). Predictors of inadequate bowel preparation for inpatient colonoscopy. Turk J Gastroenterol.

[14] Tariq H, Kamal MU, Sapkota B (2019). Evaluation of the combined effect of factors influencing bowel preparation and adenoma detection rates in patients undergoing colonoscopy. BMJ Open Gastroenterol.

[15] American Society of Anesthesiologists. ASA Physical Status Classification System,. 2020 https://www.asahq.org/standards-and-guidelines/asa-physical-status-classification-system. Accessed 10 Feb 2022.

[16] Kastenberg D, Bertiger G, Brogadir S (2018). Bowel preparation quality scales for colonoscopy. World J Gastroenterol.

[17] Johnson DA, Barkun AN, Cohen LB (2014). Optimizing adequacy of bowel cleansing for colonoscopy: recommendations from the US multi-society task force on colorectal cancer. Gastroenterology.

[18] Ergen WF, Pasricha T, Hubbard FJ (2016). Providing hospitalized patients with an educational booklet increases the quality of colonoscopy bowel preparation. Clin Gastroenterol Hepatol.

[19] Grassini M, Verna C, Niola P, Navino M, Battaglia E, Bassotti G (2007). Appropriateness of colonoscopy diagnostic yield and safety in guidelines. World J Gastroenterol.

[20] Liu A, Yan S, Wang H (2020). Ward nurses-focused educational intervention improves the quality of bowel preparation in inpatients undergoing colonoscopy A CONSORT-compliant randomized controlled trial. Medicine (Baltimore).

[21] Shah-Khan SM, Cumberledge J, Reynolds GJ (2017). Using the plan-do-study-act approach to improve inpatient colonoscopy preparation. BMJ Open Qual.

